# Fabrication of Ordered Nanopattern by using ABC Triblock Copolymer with Salt in Toluene

**DOI:** 10.1186/s11671-017-2260-0

**Published:** 2017-08-15

**Authors:** Hailiang Huang, Benbin Zhong, Xihong Zu, Hongsheng Luo, Wenjing Lin, Minghai Zhang, Yazhou Zhong, Guobin Yi

**Affiliations:** School of Chemical Engineering and Light Industry, Guangdong University of Technology, Guangzhou, Guangdong 510006 People’s Republic of China

**Keywords:** Ultrasound, Microphase separation, Triblock copolymer, Lithium chloride

## Abstract

Ordered nanopatterns of triblock copolymer polystyrene-*block*-poly(2-vinylpyridine)-*block*- poly (ethylene oxide)(PS-*b*-P2VP-*b*-PEO) have been achieved by the addition of lithium chloride (LiCl). The morphological and structural evolution of PS-*b*-P2VP-*b*-PEO/LiCl thin films were systematically investigated by varying different experimental parameters, including the treatment for polymer solution after the addition of LiCl, the time scale of ultrasonic treatment and the molar ratio of Li^+^ ions to the total number of oxygen atoms (O) in PEO block and the nitrogen atoms (N) in P2VP block. When toluene was used as the solvent for LiCl, ordered nanopattern with cylinders or nanostripes could be obtained after spin-coating. The mechanism of nanopattern transformation was related to the loading of LiCl in different microdomains.

## Background

Recently, ion/block copolymers (BCPs) hybrids have become highly attractive materials due to their flexibility, process stability, self-assembling ability and novel features of inorganic components such as electronic, magnetic and optical properties [1–3]. Spatz and co-workers created fused silica substrates with nanopillars on both sides with 99.8% transmittance and 0.02% reflectance, which was helpful for many laser applications [4]. Black et al. fabricated densely packed silicon nanotextures with feature sizes smaller than 50 nm by block copolymer self-assembly to enhance the broadband antireflection of solar cells [5]. Morris et al. fabricated Si nanowire array by self-assembly of block copolymer with LiCl, which showed the possible application in the area of photonics and photoluminescence [6].

Compared with diblock copolymers (diBCPs), ABC triblock copolymer (triBCPs) can assemble into new morphologies such as periodic arrays of core/shell spheres and cylinders, tetragonal lattices of cylinders, and bicontinuous and tricontinuous ordered mesophases [7–15]. However, ion/triBCPs hybrids are rarely reported [16]. To further explore the novel properties of ABC triBCPs and develop more performance requirements, it is necessary to study the ion/triBCPs hybrids.

The addition of salts into the BCPs is one of effective way to obtain ordered nanopatterns. Researchers have found that polyethylene oxide (PEO) [17–19], polymethyl methacrylate (PMMA) [20], poly(ε-caprolactone) (PCL) [21] or polyvinyl pyridine (PVP) [22, 23] are ion-dissolving blocks, and polystyrene (PS) [24] is a non-conducting block. Wang and co-workers suggested that the selection of metal ions to blocks was primarily due to the large solvation energy when the lithium salts associate with the polar PEO domains, leading to a large increase in the effective segregation strength with lithium salt loading [25, 26].

In previous experiments [6, 17, 27], the co-solvents for salts are frequently used because of the solubility of salts and the efficiency of coordination between salts and BCPs. Russell et al. continuously stirred after the mixture of LiCl in tetrahydrofuran (THF) and polystyrene-*block*-poly(methyl methacrylate) (PS-*b*-PMMA) toluene solution with moderate heating until most of THF was evaporated and the solutions became clear. And they spent a great deal of time (about 24 h) on stir and post-treatment (solvent vapor annealing and thermal annealing) to obtain ordered microphase-separated nanostructure [17, 28].

Herein, we demonstrated a simple and convenient approach to generate various ordered nanopatterns of ion/triBCPs hybrids by spin-coating method without any further treatments. Morphological and structural variations of PS-*b*-P2VP-*b*-PEO thin films with different salt concentrations were examined by adjusting various processing parameters. This work indicated that the coordination between PS-*b*-P2VP-*b*-PEO and LiCl-toluene could be accelerated by ultrasonic treatment for fabricating ordered nanopattern.

## Methods

### Materials

Triblock copolymer polystyrene-*block*-poly(2-vinylpyridine)-*block*-poly(ethylene oxide)(PS- *b*-P2VP-*b*-PEO, 45,000 g/mol, 16,000 g/mol, 8500 g/mol, M_w_/M_n_ = 1.05) was purchased from Polymer Source Inc. and used without further purification in this study. Anhydrous lithium chloride (LiCl, 95%+, AR) was purchased from Tianjin Fuchen Chemical Reagents Factory. Toluene (99 + %), ethanol and N,N-Dimethylform amide (DMF, analytical grade) were purchased from Tianjin Damao Chemical Co. Ltd. Silicon(Si) wafer was purchased from No.46 Research Institute of China Electronics Technology Group Corporation (CETC).

### Sample Preparation

Si wafers were cleaned in DMF, ethanol and deionized water under ultrasonic for 30 min at room temperature, respectively. 0.1 wt% PS-*b*-P2VP-*b*-PEO toluene solution was stirred for 24 h at room temperature. And LiCl was dispersed in toluene by ultrasound for 30 min at room temperature. Then various volume of LiCl toluene solution was immediately added to the PS-*b*-P2VP-*b*-PEO micellar solutions. Those mixtures were treated by different ways to trigger complexation between Li^+^ ions and polymer chains. The resultant solutions were spin-coated immediately onto the substrate at 3000 rpm for 1 min after filtration. At last, the films were dried under nitrogen at room temperature to remove the residual solvent.

### Characterization

Atomic force microscope (AFM) in SCANASYST-AIR mode (Nanoscope-V Multimode 8, Bruker Inc., Germany) by using a silicon cantilever (spring constant 5 N/m and resonant frequency ~ 150 kHz, Budget Sensors, Bulgaria Ltd.) was used to investigate the morphological features of PS-*b*-P2VP-*b*-PEO thin films. High-resolution transmission electron microscopy (HRTEM) measurement was carried out on a JEM-2100HR (JEOL, Japan) operated at 200 kV accelerating voltage. Film samples for TEM were prepared onto carbon-coated copper grids. Those samples were exposed to I_2_ vapor for certain time period. Fourier transform infrared (FT-IR) spectra were recorded with a Nicolet 6700 (Thermo, USA) spectrophotometer in the range of 4000–400 cm^−1^ with KBr plates. Ultraviolet–visible (UV-vis) spectra were obtained on a UV-2450(Shimadzu, Japan) spectrophotometer. X-ray photoelectron spectroscopy (XPS) measurements were performed on ESCALAB 250 (Thermo, USA) with Al Ka excitation.

## Results and Discussion

### Morphology of Pure PS-*b*-P2VP-*b*-PEO Thin Film

**Fig. 1:**
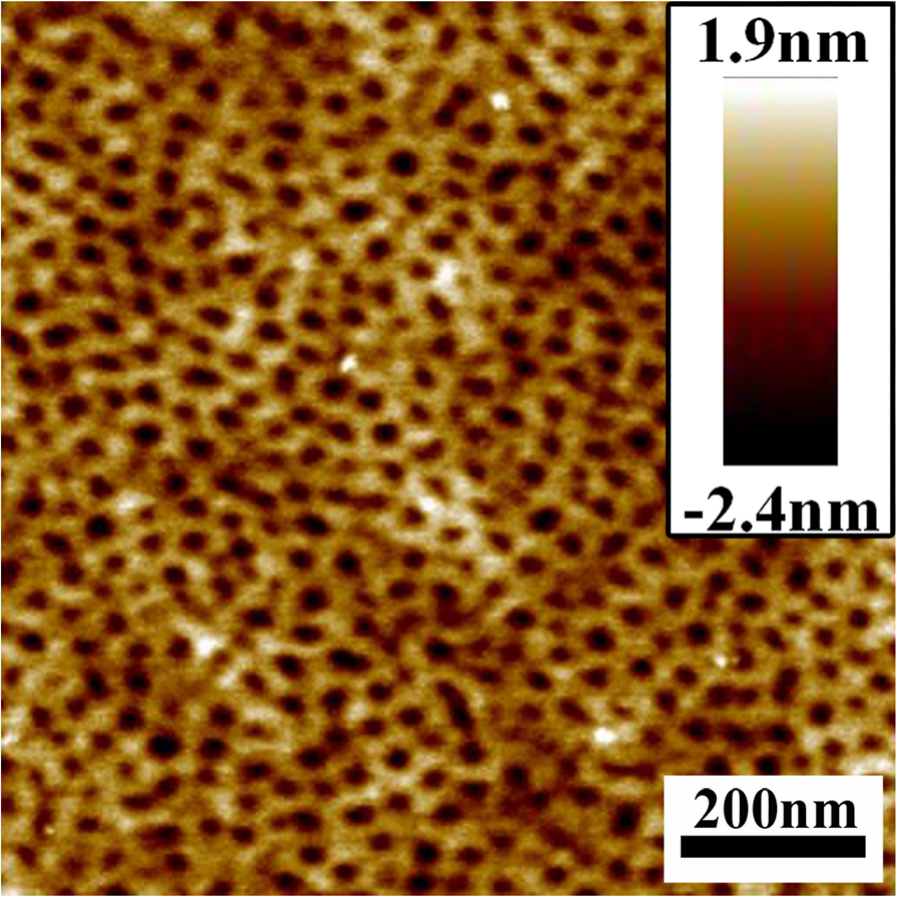
AFM height images of PS-*b*-P2VP-*b*-PEO films spin-coated from 0.1 wt% PS-*b*-P2VP-*b*-PEO toluene solution

When 0.1 wt% PS-b-P2VP-b-PEO toluene solution was stirred for 24 h and spin-coated on silicon wafer, nanoporous patterns could be observed in Fig. 1. The average size of nanopores was about 22 nm.

#### Dispersion of LiCl in Toluene

**Fig. 2:**
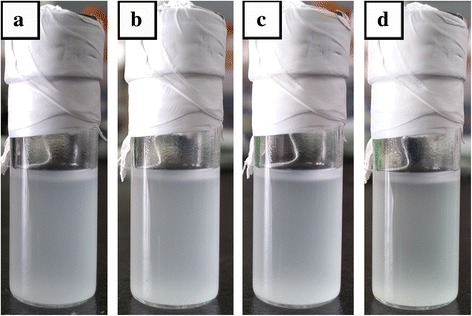
Dispersion of LiCl in toluene after ultrasonic treatment without and with different aging time: (**a**) without aging time, (**b**) 1 min, (**c**) 3 min, (**d**) 5 min

Dispersions of LiCl in toluene with various aging times are shown in Fig. 2. Toluene was not a good solvent for LiCl. So suspension with unstable status could be seen after ultrasonic treatment (Fig. 2a). It was noticeable that little sedimentation phenomenon was observed when the aging time was 5 min (Fig. 2d). Therefore, the prepared suspension should be used immediately after ultrasonic treatment.

#### Effect of Methods to Trigger the Coordination between LiCl and Polymer Chains

**Fig. 3:**
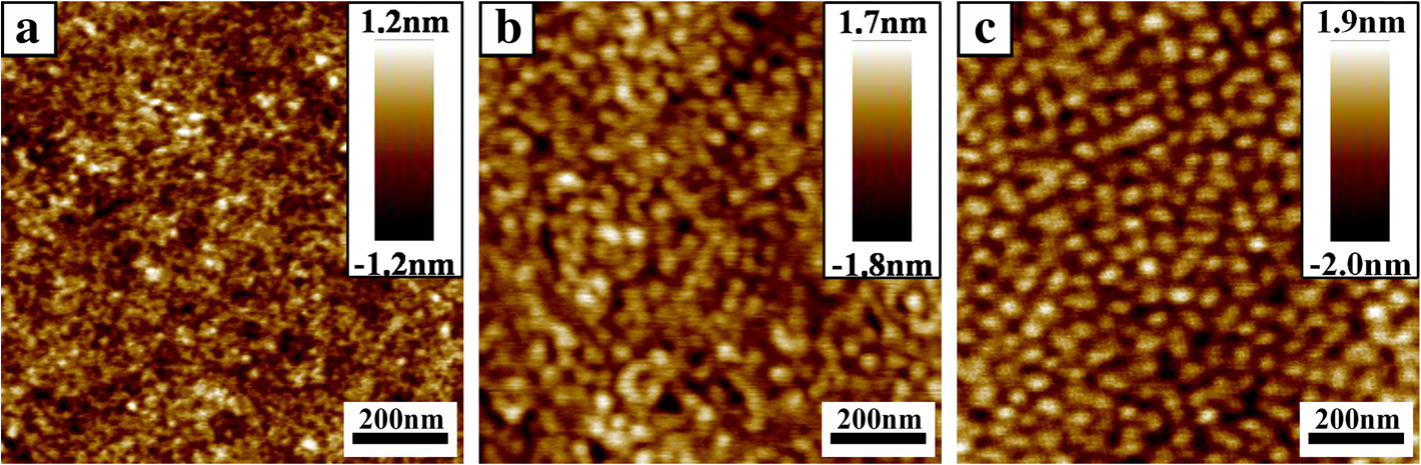
AFM height images of PS-*b*-P2VP-*b*-PEO films spin-coated from 0.1 wt% toluene solution with different methods after the addition of LiCl-toluene when the molar ratio of Li^+^ ions to the total number of oxygen atoms (O) in PEO block and the nitrogen atoms (N) is 1:32.2: (**a**) 1500 rpm stirring for 30 min at room temperature, (**b**) 1500 rpm stirring for 30 min at 75 °C, (**c**) ultrasonic treatment for 30 min at room temperature

Generally, stir and post-treatment are required for polymer solution containing metal salts in order to trigger the coordination between salts and polymer chains for fabrication of ordered nanostructure, which takes a lot of time [22, 28]. And the ultrasound is the simple way to accelerate the coordination between metal ions and block copolymer [29–31]. In order to demonstrate the advantage of ultrasonic treatment in this work, different methods were used after the mix of LiCl-toluene and triblock copolymer solution when the molar ratio of Li^+^ ions to the total number of oxygen atoms (O) in PEO block and the nitrogen atoms (N) was 1:32.2([Li^+^]:[O + N] = 1:32.2). When the mixed solution was stirred (1500 rpm) for 30 min at room temperature and then spin-coated onto substrate, no distinct ordered structure was observed in Fig. 3a. When the mixed solution was stirred at 1500 rpm for 30 min at 75 °C and then spin-coated onto substrate, disordered cylindrical microdomains appeared in Fig. 3b. When the mixed solution was placed in ultrasonic cleaner for 30 min at room temperature, microphase-separated nanopattern with cylindrical microdomain was obtained obviously in Fig. 3c after spin-coating. The energy provided by sound waves was able to disrupt the larger aggregates of the micelles. And the sound waves could further increase the diffusion rate of metal ions in the solution, so the loading of Li^+^ ions in micelles were expected to happen much faster than the conventional stirring method. This result indicated that ultrasonic treatment was a useful method to improve the efficiency of coordination between Li^+^ ions and polymer chains.

#### Effect of Time Scale

**Fig. 4:**
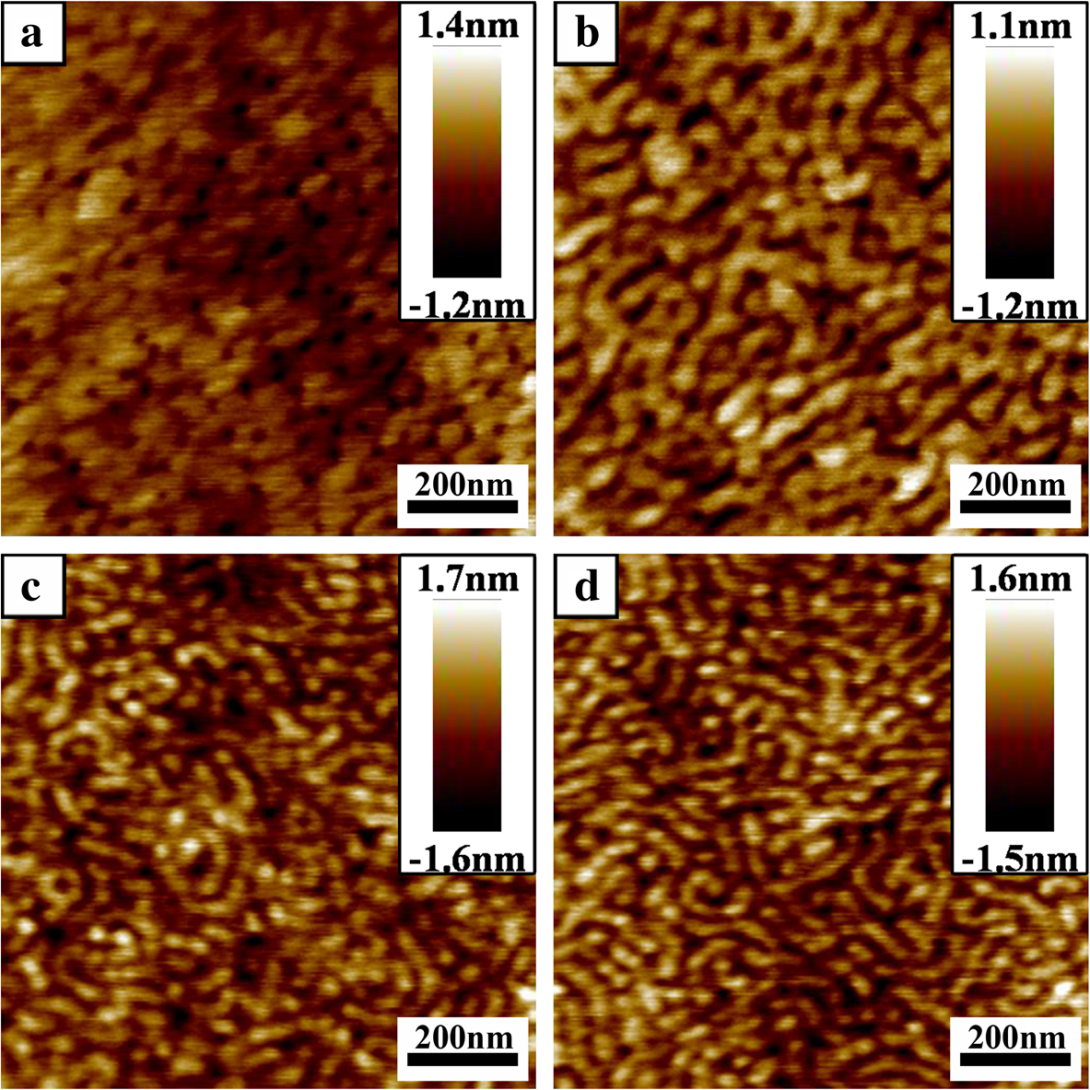
AFM height images of PS-*b*-P2VP-*b*-PEO films spin-coated from 0.1 wt% polymer-LiCl toluene solution with various time scale of ultrasonic treatment when the molar ratio of Li^+^ ions to the total number of oxygen atoms (O) in PEO block and the nitrogen atoms (N) is 1:32.2: (**a**) 7.5 min, (**b**) 15 min, (**c**) 22.5 min, (**d**) 37.5 min

In order to investigate the time scale of ultrasonic treatment, the mixed solution ([Li^+^]:[O + N] = 1:32.2) was placed in ultrasonic cleaners for various times before spin-coating. When the time was 7.5 min (Fig. 4a), the nanoporous morphology was similar to the film in Fig. 1. Compared with the film in Fig. 1, the number and the average size of nanopores decreased, which indicated that Li^+^ ions began to load in PS-*b*-P2VP-*b*-PEO polymer chains after 7.5 min. The Li^+^ ions loaded in polymer chains would increase with the time increasing. Parts of nanopores were connected when the time increased to 15 min (Fig. 4b). When the time was 22.5 min, the nanopattern exhibited a coexistence of nanostripes and cylinders (Fig. 4c). When the time was prolonged to 30 min, microphase-separation with cylindrical microdomains occurred obviously (Fig. 3c). As the time extended to 37.5 min, the coexistence of nanostripes and cylindrical microdomains appeared again (Fig. 4d). From above results, when the time was less than 30 min, the complexation between Li^+^ ions and PS-*b*-P2VP-*b*-PEO was accelerated by ultrasonic treatment so that more and more Li^+^ ions were coordinated with PS-*b*-P2VP-*b*-PEO, resulting in transition of nanopattern from nanoporous array to cylindrical array. When the time was more than 30 min, the energy provided by sound waves would break the coordination of Li^+^ ions and polymer chains so that disordered nanopattern was found instead of the cylindrical array. Therefore, the time of ultrasonic treatment should be controlled in appropriate range to obtain obvious microphase-separated nanopattern.

#### Effect of LiCl Content in PS-*b*-P2VP-*b*-PEO Thin Films

**Fig. 5:**
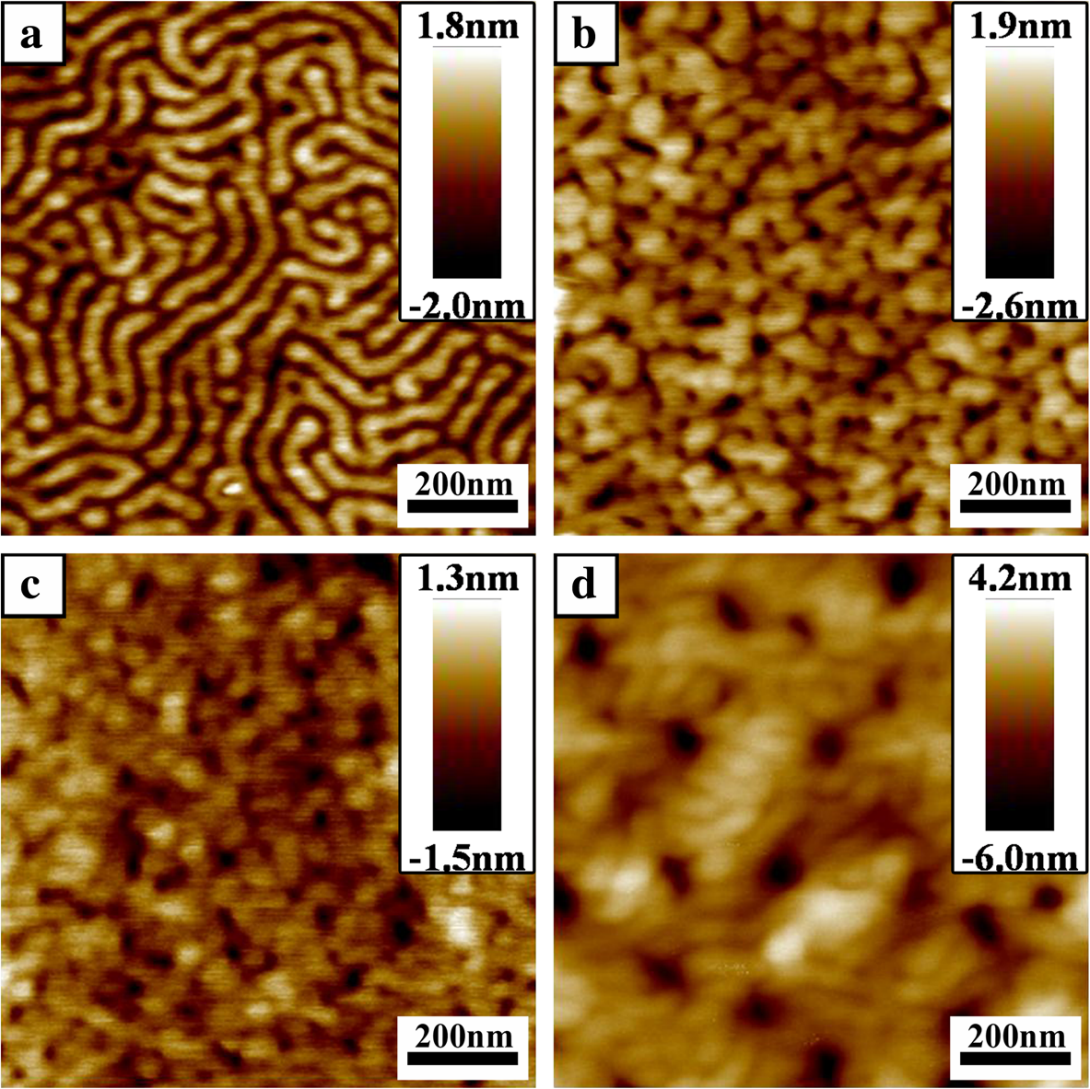
AFM height images of PS-*b*-P2VP-*b*-PEO films spin-coated from 0.1wt%polymer-LiCl toluene solution with various molar ratio of Li^​+^ ions to ethylene oxide moieties and pyridine groups: (**a**) 1:40.25, (**b**) 1:24.15, (**c**) 1:16.1, (**d**) 1:8.05

The addition of LiCl has significant effects on morphology since Li^+^ ions could be loaded in P2VP and PEO blocks [17–19, 22, 23]. And the molar ratio ([Li^+^]:[O + N]) was varied in our work (Fig. 5).

When the molar ratio was 1:40.25, the nanopattern of stripes was obtained (Fig. 5a). When the molar ratio decreased to 1:32.2, nanopattern with cylindrical microdomains could be seen in Fig. 3c. As the molar ratio was 1:24.15, a lot of nanopores were connected to show the tendency from nanopores pattern transform to nanostripes (Fig. 5b). When the molar ratio was 1:16.1, disordered nanopores become the overall morphology (Fig. 5c). The average size of holes was larger than the film in Fig. 1. As the molar ratio further decreased to 1:8.05, a few of nanopaores was observed in Fig. 5d. The average diameter of these pores was more than 40 nm. From above results, an order-to-disorder transition was shown in Fig. 5 by controlling the addition of LiCl in ion/polymer hybrids. The change of LiCl loaded in polymer chains was the reason for the morphological transition. The LiCl loading in polymer chains increased with decreasing molar ratio ([Li^+^]:[O + N]), leading to the different phase behaviors of the PS-*b*-P2VP-*b*-PEO/LiCl hybrids. And the ordered arrangements of PS-*b*-P2VP-*b*-PEO/LiCl hybrids were formed with the critical amount of LiCl loaded.

### Microdomains Location of Three Blocks in PS-*b*-P2VP-*b*-PEO Thin Films

In order to explore the microdomain location of the three blocks in PS-*b*-P2VP-*b*-PEO thin film under different conditions, those samples were exposed to I_2_ vapor for certain period before TEM measurement.

**Fig. 6:**
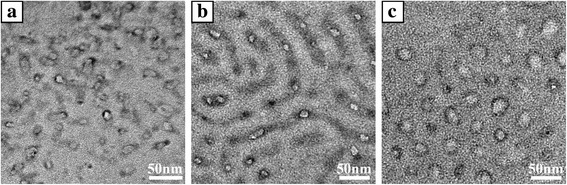
TEM images of PS-*b*-P2VP-*b*-PEO film after I_2_ staining with and without LiCl: (**a**) without LiCl, (**b**) with LiCl-toluene and the molar ratio of LiCl to ethylene oxide moieties and pyridine groups was 1:40.25, (**c**) with LiCl-toluene and the molar ratio of LiCl to ethylene oxide moieties and pyridine groups was 1:32.2

The PS-*b*-P2VP-*b*-PEO thin film without LiCl exhibited an array of dark rings after the selective staining of P2VP blocks, indicating that the periphery of the hole corresponded to P2VP blocks (Fig. 6a). Thus, the rest of the hole should match with PEO blocks. The continuous matrix was PS blocks. The average outer diameter of the dark rings was about 21 nm and the average inner diameter of the dark rings was about 16 nm.

When the molar ratio ([Li^+^]:[O + N]) was 1:40.25 after I_2_ selective staining, the nanopattern of stripes was obtained (Fig. 6b). The bright regions of the spheres were depressed in striated structure. The bright regions were PEO blocks and the rest of stripes were P2VP microdomains. Hence, the continuous matrix was PS blocks. Distinct dark particulates (of LiOH presumably) were observed in P2VP domains [32]. The average diameter of PEO domains was about 17 nm, which was similar to the average domain size of PEO blocks in Fig. 6a. And the P2VP domains transformed from dark rings to stripes. This result indicated that most of Li^+^ ions were preferentially coordinated with P2VP blocks when the molar ratio was 1:40.25.

When the molar ratio ([Li^+^]:[O + N]) decreased to 1:32.2, an array of dark rings could also be seen (Fig. 6c) after I_2_ selective staining. The dark rings were P2VP microdomains and the bright regions were PEO blocks. The continuous matrix was PS blocks. The average outer diameter of dark rings was about 32 nm, and the average inner diameter of dark ring was about 26 nm. It was demonstrated that the cylindrical domains in Fig. 3c were core-shell structure. The outer shell was P2VP blocks and the core was PEO blocks. Compared with the film in Fig. 6a, the PEO microdomains were obviously swelled and the P2VP ﻿domains slightly increased. Compared with the film in Fig. 6b, this result indicated that more Li^+^ ions were coordinated with PEO blocks with more LiCl in PS-b-P2VP-b-PEO thin film.

**Fig. 7:**
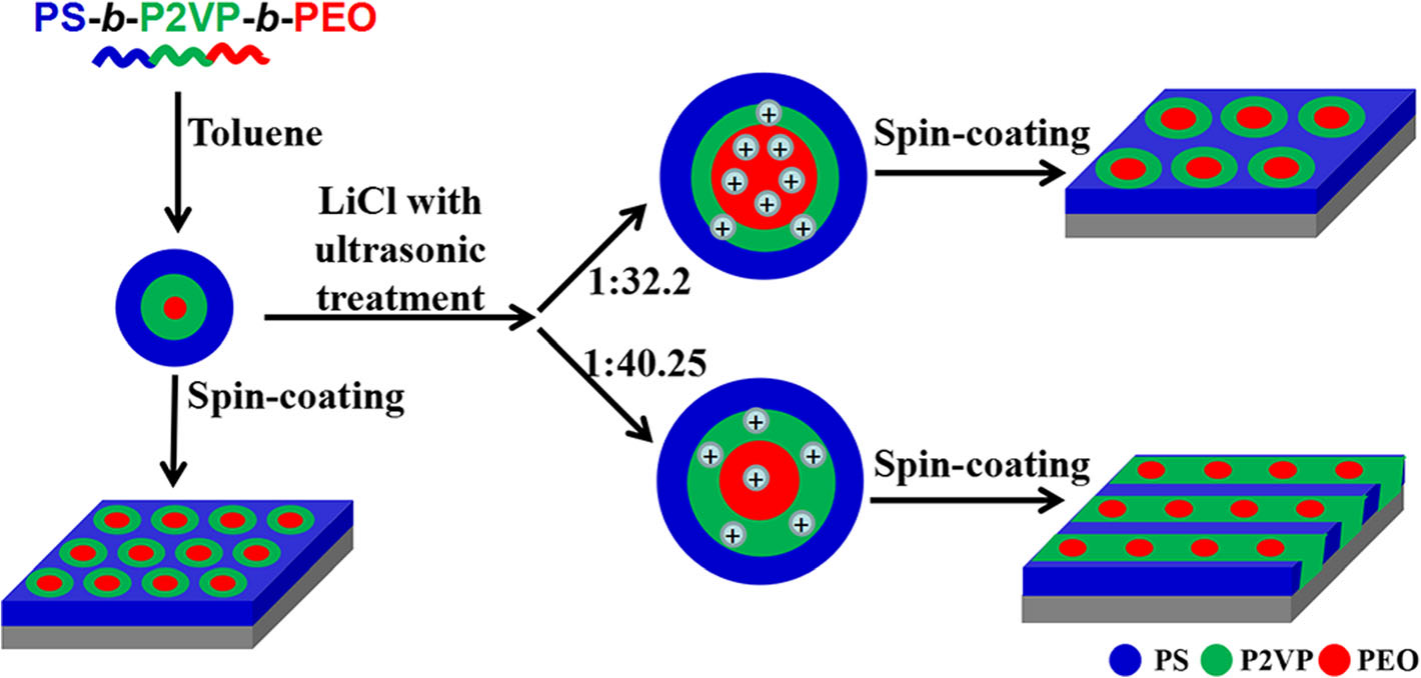
Schematic illustration of the fabrication of PS-*b*-P2VP-*b*-PEO nanopattern with and without Li^+^ ions

The difference of Fig. 6b, c can be explained as shown in Fig. 7. Because of the selectivity of toluene for three blocks, the nanostructure of PS-*b*-P2VP-*b*-PEO micelles in toluene was core-shell structure. Considering the sequence of the three blocks in PS-*b*-P2VP-*b*-PEO, PS blocks were the outer shell. The inner shell was P2VP domain and the core was PEO blocks. When the molar ratio ([Li^+^]:[O + N]) was 1:40.25, the Li^+^ ions were mainly focused on the inner shell of P2VP blocks because of the limited content of LiCl and the resistance of P2VP inner shell. As a result, only a few of Li^+^ ions were coordinated with PEO microdomains. When the molar ratio ([Li^+^]:[O + N]) was 1:32.2, the interaction parameter of Li^+^ ions and the PEO blocks effectively increased due to the increase of LiCl-toluene, resulting in the obvious swelling in PEO domains [32–36].

### Analysis of Competitive Interactions of Li^+^ ions with PEO and P2VP Blocks

**Fig. 8:**
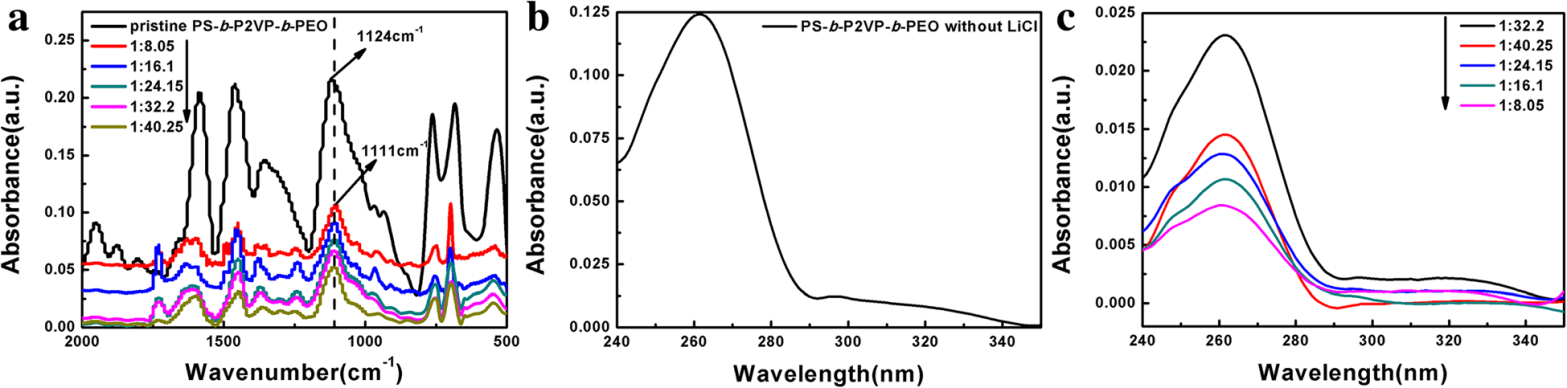
(**a**) FT-IR spectra of pure PS-*b*-P2VP-*b*-PEO thin film and the thin film with LiCl-toluene; (**b**) UV-vis spectra of pure PS-*b*-P2VP-*b*-PEO thin film; (**c**) UV-vis spectra of PS-*b*-P2VP-*b*-PEO thin film with LiCl-toluene

It is noteworthy that the competitive interactions of Li^+^ ions with both the PEO and P2VP blocks exist in PS-*b*-P2VP-*b*-PEO/LiCl hybrids [3]. The interaction between the Li^+^ ions and the PEO blocks was characterized by FT-IR (Fig. 8a). The parameter of *I*_a_/*I*_f_, which was the ratio of the peak intensity corresponding to the associated C-O-C to the peak intensity of free C-O-C, was used to evaluate the coordination between the Li^+^ ions and the PEO blocks (Table 1) [37, 38]. The C-O-C stretching vibration changed from 1124 to 1111 cm^−1^. The value of *I*_a_/*I*_f_ increased with the doped LiCl increasing, indicating that the loading of Li^+^ ions in PEO blocks increased when the molar ratio ([Li^+^]:[O + N]) decreased from 1:40.25 to 1:8.05.

**Table 1:** The data of *I*_a_/*I*_f_ for different PS-*b*-P2VP-*b*-PEO/LiCl hybrids in LiCl-toluene

[Li^+^]:[O + N]	*I*_a_/*I*_f_
1:40.25	0.2522
1:32.2	0.3165
1:24.15	0.3670
1:16.1	0.4266
1:8.05	0.4908

The UV-vis spectra of various PS-*b*-P2VP-*b*-PEO thin films are illustrated in Fig. 8b, c. The absorption peak at 262 nm was assigned to pyridine groups and phenyl groups of PS-*b*-P2VP-*b*-PEO [39]. Based on the previous study [24], the obvious change of the intensity was attributed to the coordination between Li^+^ ions and pyridine groups. The intensities of absorption peak at 262 nm with different samples were summarized in Table 2. The intensities of absorption peak at 262 nm for PS-*b*-P2VP-*b*-PEO thin films with LiCl (Fig. 8c) were weaker than the pure film (Fig. 8b). When the molar ratio ([Li^+^]:[O + N]) was 1:40.25, 1:24.15, 1:16.1 and 1:8.05, the intensity of absorption peak at 262 nm decreased with the LiCl addition increasing (Fig. 8c), indicating that more and more Li^+^ ions were coordinated with the P2VP blocks and PEO blocks﻿. However, when the molar ratio ([Li^+^]:[O+N]) was 1:32.2, the absorption peak at 262 nm was stronger than the molar ratio 1:40.25. The reason should be that most of Li+ ions were loaded in PEO blocks but not P2VP blocks when the molar ratio was about 1:32.2, and the least LiCl was loaded in P2VP blocks at this molar ratio ([Li^+^]:[O + N] = 1:32.2) compared with other thin films with LiCl.

**Table 2:** The intensity of absorption peak at 262 nm with different PS-*b*-P2VP-*b*-PEO thin films

Samples	Intensity at 262 nm (a.u.)
PS-*b*-P2VP-*b*-PEO thin film	0.123
1:32.2	0.023
1:40.25	0.015
1:24.15	0.013
1:16.1	0.011
1:8.05	0.009

**Fig. 9:**
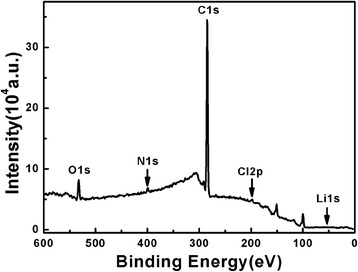
XPS survey spectra of PS-*b*-P2VP-*b*-PEO thin film with LiCl-toluene

**Fig. 10:**
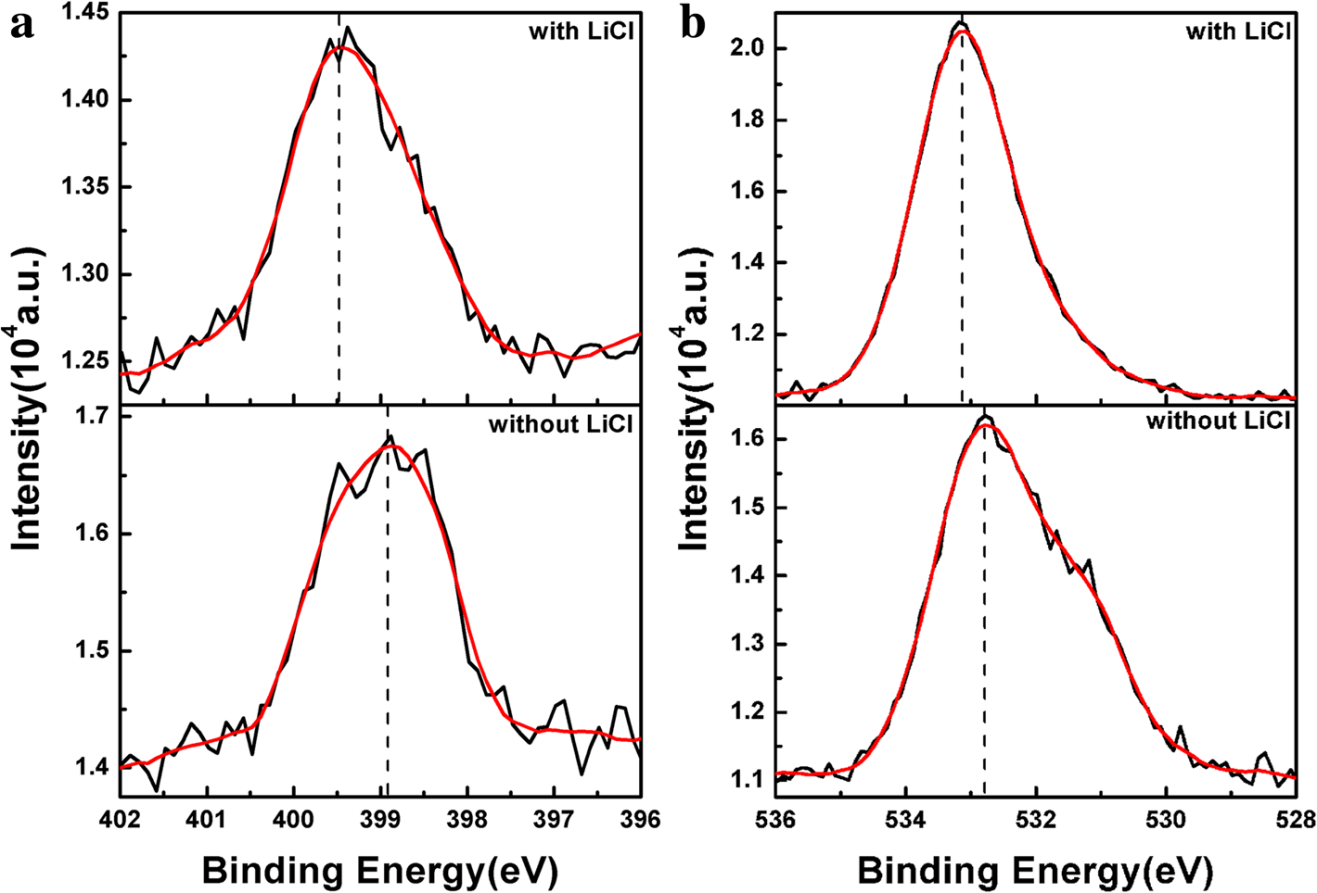
High-resolution XPS spectra of (**a**) N1 s binding energy and (**b**) O1s binding energy in PS-*b*-P2VP-*b*-PEO with and without LiCl-toluene

The PS-*b*-P2VP-*b*-PEO thin films without and with LiCl-toluene ([Li^+^]:[O + N] = 1:32.2) were analyzed by X-ray photoelectron spectroscopy (XPS) (Figs. 9 and 10). XPS survey spectra (Fig. 9) of PS-*b*-P2VP-*b*-PEO with LiCl confirmed the presence of C, O, N, Li and Cl. The C1s binding energy in C-C bonds was 284.78 eV. The O1s binding energy of C-O-C in PEO block was 533.08 eV, and N1 s binding energy based on the P2VP block was 399.48 eV. The Cl2p appeared at 198.28 eV, and Li1s appeared in 55.88 eV. High resolution XPS spectra of N1 s binding energy and O1s binding energy in PS-*b*-P2VP-*b*-PEO with and without LiCl were shown in Fig. 10a, b. The N1 s binding energy in PS-*b*-P2VP-*b*-PEO without LiCl was 398.88 eV, but the binding energy in the thin film with LiCl was 399.48 eV. The O1s binding energy in PS-*b*-P2VP-*b*-PEO without LiCl was 532.78 eV, but the binding energy in the thin film with LiCl was 533.08 eV. These shifts in binding energy were consequences of electron withdrawing effect caused by the coordination between Li^+^ and PS-*b*-P2VP-*b*-PEO [40], validating the presence of Li element in the thin film after Li^+^ ions were loaded. These results were essentially identical to the results in Fig. 8.

## Conclusions

In this study, we present a simple approach to fabricate ordered nanopatterns of ion/triblock copolymers hybrids without post-processing. This work demonstrated that toluene could be used as co-solvents for LiCl in short time. An order-to-disorder transition was triggered by varying the addition of LiCl-toluene with ultrasonic treatment. And ordered microphase-separated nanopatterns of cylindrical array and stripes were obtained. The mechanism of the morphological transition was due to the LiCl loaded in different ion-dissolving blocks. This rapid synthesis might boost future studies of ion/triblock copolymers hybrids because of the advantage of ultrasonic as compared to the conventional routes. Furthermore, this approach has potential applications in developing ultra-small devices via techniques such as pattern transfer owing to its simplicity, effectiveness and low cost, especially regarding to fabrication time.

## Abbreviations

[Li^+^]:[O + N]: the molar ratio of Li^+^ ions to the total number of oxygen atoms (O) in PEO block and the nitrogen atoms (N)
AFM: Atomic force microscope
CETC: China Electronics Technology Group Corporation
diBCPs: diblock copolymers
DMF: N,N-Dimethylform amide
FT-IR: Fourier transform infrared
HRTEM: High resolution transmission electron microscopy
LiCl: lithium chloride
LiOH: Lithium hydroxide
PCL: poly(ε-caprolactone)
PMMA: polymethyl methacrylate
PS: polystyrene
PS-*b*-P2VP-*b*-PEO: polystyrene-*block*-poly(2-vinylpyridine)-*block*-poly(ethylene oxide)
PVP: polyvinyl pyridine
triBCPs: triblock copolymers
UV-vis: Ultraviolet–visible
XPS: X-ray photoelectron spectroscopy
